# The Comparative Studies of Binding Activity of Curcumin and Didemethylated Curcumin with Selenite: Hydrogen Bonding vs Acid-Base Interactions

**DOI:** 10.1038/srep17614

**Published:** 2015-12-04

**Authors:** Jiahn-Haur Liao, Tzu-Hua Wu, Ming-Yi Chen, Wei-Ting Chen, Shou-Yun Lu, Yi-Hsuan Wang, Shao-Pin Wang, Yen-Min Hsu, Yi-Shiang Huang, Zih-You Huang, Yu-Ching Lin, Ching-Ming Chang, Fu-Yung Huang, Shih-Hsiung Wu

**Affiliations:** 1 Institute of Biological Chemistry, Academia Sinica, Taipei 11529, Taiwan; 2 Department of Clinical Pharmacy, School of Pharmacy, College of Pharmacy, Taipei Medical University, Taipei 110, Taiwan; 3 General Education Center, National Taipei University of Nursing and Health Sciences, Taipei, Taiwan; 4 Department of Chemistry, National Cheng Kung University, Tainan 701 Taiwan; 5 Institute of Biochemical Sciences, National Taiwan University, Taipei 10617, Taiwan

## Abstract

In this report, the *in vitro* relative capabilities of curcumin (CCM) and didemethylated curcumin (DCCM) in preventing the selenite-induced crystallin aggregation were investigated by turbidity tests and isothermal titration calorimetry (ITC). DCCM showed better activity than CCM. The conformers of CCM/SeO_3_^2−^ and DCCM/SeO_3_^2−^ complexes were optimized by molecular orbital calculations. Results reveal that the selenite anion surrounded by CCM through the H-bonding between CCM and selenite, which is also observed via IR and NMR studied. For DCCM, the primary driving force is the formation of an acid-base adduct with selenite showing that the phenolic OH group of DCCM was responsible for forming major conformer of DCCM. The formation mechanisms of selenite complexes with CCM or DCCM explain why DCCM has greater activity than CCM in extenuating the toxicity of selenite as to prevent selenite-induced lens protein aggregation.

Selenium (Se) was first discovered in early eighties and named after the Greek word, *Selene*, which means the moon^1^. The essential trace element selenium is a crucial cofactor in endogenous anti-oxidative systems of the human body. Selenium incorporates into many selenoproteins^2^ including glutathione peroxidases and thioredoxin reductases, which have important antioxidant and detoxification functions. The essentiality of selenium is further highlighted by high incidence of Kashin-Beck disease and Keshan disease in selenium deficient area^3^,^4^. Moreover, recent studies suggest that selenium has a potential in cancer prevention and can be used in cancer treatment^5^,^6^,^7^. Currently, common dietary selenium compounds include selenite, selenate, selenomethionine, methylselenocysteine and selenocystine. Among these selenium compounds, inorganic species, selenite and selenate, were most often used in nutritional supplementation. Selenate would further metabolized into selenite in a living system^8^. The main inorganic selenium compound used in most studies of cancer treatment is sodium selenite^9^,^10^,^11^,^12^,^13^,^14^,^15^. Selenite is suggested more effective antioxidants than sodium selenate or sodium selenide^16^ and to have biphasic action. Therefore, intake of selenium higher than the upper limit range (350–400 μg/day) would lead to selenium poison or selenosis^10^,^11^. The cytotoxicity of selenite has been suggested to be due to the reaction with disulfide peptide or protein^15^,^17^,^18^. It is further linked to the protein aggregation disease, for example, cataract^19^,^20^,^21^. One hypothesis regarding the beginning of the cataract development suggests that selenium could oxidize the SH groups of ion transporters in the membrane that maintains calcium homeostasis^22^. Previous finding showed that selenite would induce crystallin aggregation^19^. Both calcium and selenite accumulated in lens may lead to cataract^20^,^21^.

Curcuminoids are natural yellow-orange pigments derived from the root of Curcuma longa. For centuries, curcumin [1, 7-bis (4-hydroxy-3-methoxyphenyl)-1, 6 heptadiene-3, 5-dione] (CCM, CID 969516), a spice often found in curry powder, has been used for food and in certain medicinal preparations in Asia. CCM is the active ingredient of the rhizome of the plant turmeric (Curcuma longa Linn)^23^ and the derivatives of CCM including demethoxycurcumin, bisdemethoxycurcumin were compared for their ability to scavenge or interact with various radicals^24^. Recently, the health properties (neuroprotection, chemo-, and cancer prevention) of curcuminoids have gained increasing attention^25^ including its *in vivo* anti-cataractogenesis properties^26^,^27^,^28^,^29^,^30^,^31^. Previously, curcumin has been observed to protect lenses from cataractogenesis through against various chemical insults^30^,^32^,^33^. Most of these properties have been related to the anti-oxidant activity of curcumin. An experiment showed curcumin can modulate the expression of chaperones inside lenses^34^. Another experiment demonstrated that curcumin possesses protect effect on ionizing radiation-induced cataractogenesis^35^. Efforts were on modifying structure of curcumin to enhance its anti- cataractogenesis activity^36^. There is still a lot more research needs to be done before the use of CCM as an effective anti-cataract agent for humans^37^.

Our previous study showed that astaxanthin and pirenoxine ameliorates selenite induced cataractogenesis by interacting with selenite^19^,^21^. CCM was reported to prevent or decrease selenium-induced oxidative stress leading to inhibition of lens opacification^28^. The mechanisms how selenite can induce cataract formation are extensively investigated^38^. However, it is unclear how CCM interacts with selenite. This study aimed to gain insight into the chemistry of CCM concerning its anti-cataract effect by FT-IR, ITC, NMR and computational analysis. Even though salicylidenecurcumin and benzalidenecurcumin, derivatives^36^ of CCM were shown to attenuate cataract formation *in vitro*; however, the activity of demethylated compounds in anti-cataract is unknown. To compare with CCM, didemethylated curcumin (DCCM, CID 5469425) were also investigated with parallel methods.

## Result and Discussion

In addition to conventional uses in dye industry and food science, curcumin (CCM, see below for the molecular structure) has been found to possess versatile biomedical applications^25^,^36^,^39^,^40^,^41^. In order to get insight into the chemical nature of those observations, quantum mechanics studies have been made to refine its practical uses^41^,^42^, including those to explore the potential of CCM in non-linear optical applications^43^. One of the reported medicinal applications motivates this work is that CCM and its derivatives would attenuate selenite-induced cataract formation^36^. In combination with chelating metal-ions exhibited by CCM^5^,^6^, it is obvious that CCM can form complex with both cation and selenite, which is similar to PRX anion^20^. With the aim to understand the nature of published biological properties exhibited by CCM, the binding mechanisms have been studied by density functional theory (DFT) calculations^44^. From the in silico experiment, we found that the methyl group on curcumin may play important roles in binding selenite. To investigate the roles of methyl groups, we choose DCCM as a research target. For comparative and/or supplementary purpose, the didemethylated curcumin (DCCM) have also been investigated by parallel methods. The study undertaken here demonstrates the beautiful coupling of quantum mechanics and experimental observations obtained by turbidity test and Isothermal Titration Calorimetry (ITC) experiments, as well as by solution NMR and solid-state IR spectroscopic techniques. In the NMR studies, acetonitrile (CH_3_CN) was added to improve the solubility and, simultaneously or more importantly, to get CCM structural information in the basic solutions.

**Figure 1:**
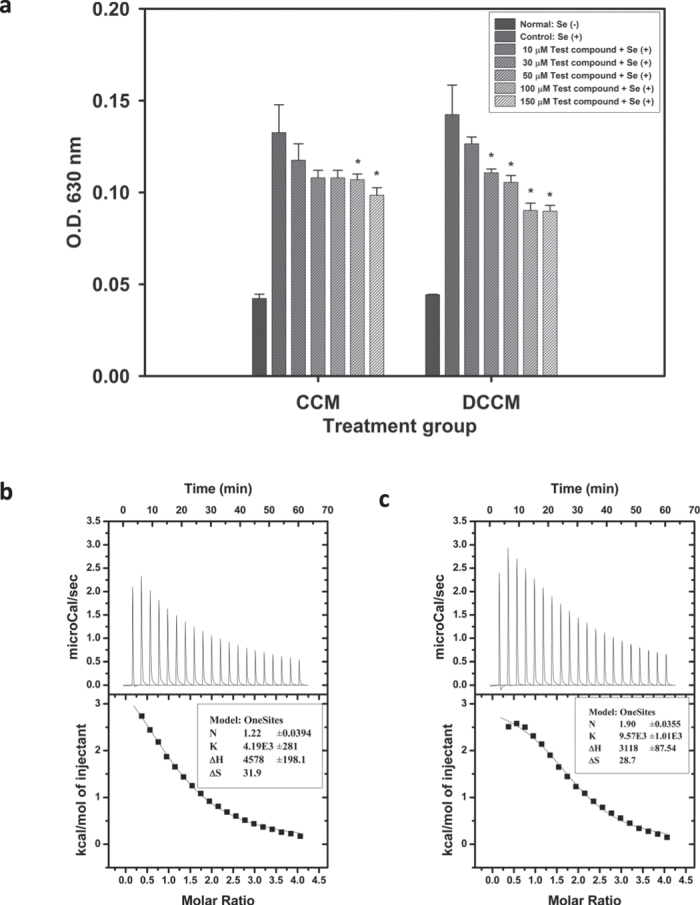
(**a**) *In vitro* lens crystallin turbidity assay of CCM and DCCM. Comparison of CCM and DCCM were performed using 10 ~ 150 μM of each compound incubated at 37 °C for four days. Column Se (+) and Se (−) indicated crystallin solution incubated with or without sodium selenite. *P < 0.05 for comparison with the Se (+) group. (**b,c**): ITC measurements for CCM and DCCM titrated with selenite, respectively.

One sees that, from Fig. 1a, DCCM possesses better activity than CCM through turbidity experiments. Our results show that 30 μM DCCM resulted in significant inhibition on turbidity formation, whereas 100 μM CCM was required to obtain inhibition. The result indicated that the removal of methyl groups enhances the activity of DCCM as compared to CCM. The ITC thermogram (see Fig. 1b,c) showed that DCCM possesses higher selenite binding affinity (K = 9.57 × 10^3^ M^−1^) than CCM (K = 4.19 × 10^3^ M^−1^). When interacting with selenite CCM revealed an endothermic titration curve with stoichiometry n = 1.22, an enthalpy-capture process (∆H = 4578 cal/mol), larger entropy property (∆S = 31.9 cal mol^-1^ K^-1^), and a negative Gibb’s free energy (∆G = −4928.3 cal/mol) in propanol/water (1/1) solution. DCCM titrated with selenite shows an endothermic titration curve with stoichiometry n = 1.90, an enthalpy-capture process (∆H = 3118 cal/mol), larger entropy property (∆S = 28.7 cal mol^-1^ K^-1^), and a negative Gibb’s free energy (∆G = −5434.6 cal/mol) in the same solution as curcumin. Those thermodynamic quantities indicated that the interactions of DCCM (or CCM) with selenite resulted in the solvent molecules leaving away from solute. The stoichiometry obtained from the two titration curves indicated approximately a 1:2 binding capability for DCCM in binding with selenite, which is consistent with the activity results.

**Figure 2:**
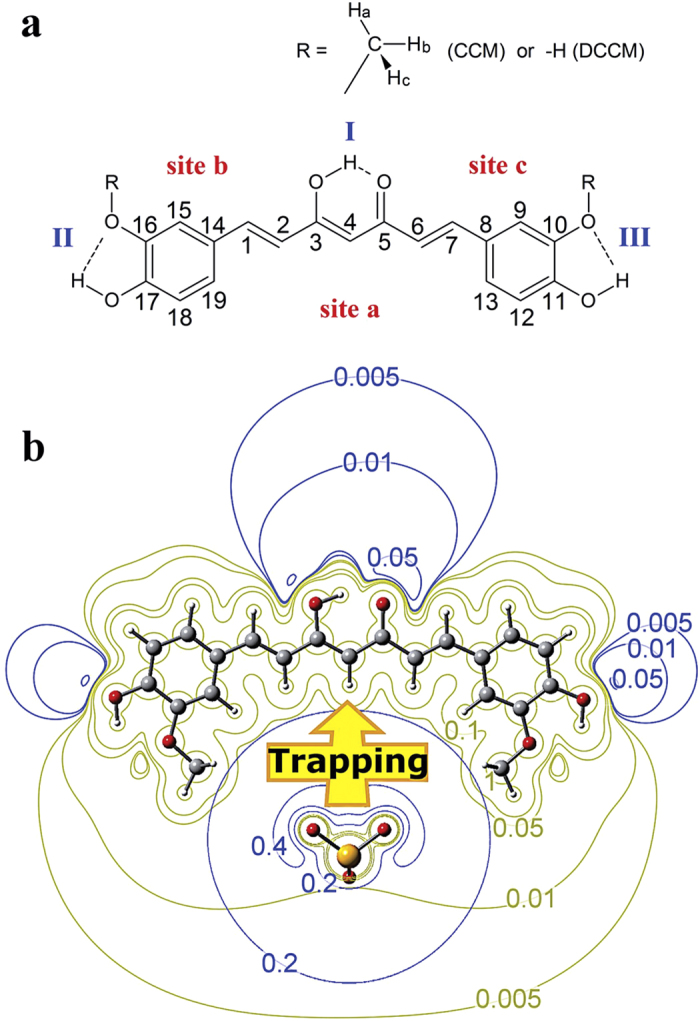
(**a**) Structure and Numbering of carbon-atoms of CCM and (**b**) Electron potential Plot and the most energy-favorable selenite trapping site of CCM/SeO_3_^2−^ adduct, denoted as CS-10 in the supporting materials. The value of E_binding_ obtained for this structure (84 kcal/mol) is distinguished larger than those of the other 12 structures (between 71 and 36 kcal/mol). The unfavorable binding at sites b and c arises from the negative electron potential governed by oxygen-atom(s).

In order to get insight into the relative effectiveness, DFT calculations have been carried out on complexes formed by CCM (or DCCM) and selenite. The numbering of CCM carbon-atoms is given in Fig. 2a. Both the keto-enol forms (denoted as A, BI, BII, and C in Figure S1) and the di-keto forms (DI-DIII) of a free CCM molecule were calculated (see Figure S1). As reported in the literature^45^, the di-keto conformers are higher in energy than the keto-enol conformers by approximately 5~7 kcal/mol in different condition (see Table S1). Intra-molecular H-bonding is a common feature found in these static conformers, which explains the reducing activity of the two phenolic hydrogens^46^. For the CCM/SeO_3_^2−^ adducts, the optimized conformers (see Figure S2 for all plausible static structures in the gas state) reveal that three sites (labelled a, b, and c in Fig. 2a) of the CCM molecule are responsible for binding SeO_3_^2−^. An analysis of the binding energies (E_binding_) of those adducts (summarized in Table S3) indicates that, for each keto-enol conformer (A, BI, BII, or C), trapping SeO_3_^2−^ at “site a” leads to a larger binding energy than binding at either site b or site c. Among the CCM/SeO_3_^2−^ adducts formed by binding SeO_3_^2−^ at “site a”, the values of E_binding_ increase in the order A(CS-1) <BI(CS-4)~BII(CS-7) <C(CS-10), as seen in Table S3. This discrimination between the keto-enol conformers can be understood based upon the electronic potential plots (EPPs) shown in Figure S3. The negatively charged keto-enol oxygens result in electronically unfavorable binding of the selenite anion at sites b or c. In contrast, positively charged hydrogen atoms form a wider chain (positioned between two phenolic hydrogens) that allows “site a” more favorably to bind SeO_3_^2−^, as indicated in Fig. 2b. It can thus be understood through the above-described trend of binding energies that the methyl hydrogen atoms can more effectively stabilize SeO_3_^2−^ if they are oriented in a way that expands the positive continuum. In two different polar solvents (water and ethanol presented in Table S3), the most energy-favored binding site is “site a”, indicating that methyl of CCM is importance for moving SeO_3_^2−^ out from protein (lower polar phase). One sees from this Table that the binding energies increase with reducing polarity of solvent ion from water to ethanol calculated values of ΔE^PCM^ for adduct CS-10 is stabilized more by 2.18 kcal/mol (other 11 adducts are more stabilized by 0.47 to 1.74 kcal/mol). Because SeO_3_^2−^ almost wrapped by CCM out of solvent, thus the interaction between SeO_3_^2−^ and solvent is great extent of loss. It implied that methyl of CCM is importance for moving SeO_3_^2−^ out from protein (lower polar phase). On other hand, real solvation condition is very complex. CCM trapping SeO_3_^2−^ accompanies with SeO_3_^2−^ releasing solvent molecules. This argument might be a plausible explanation for the entropy-driven spontaneous process concluded by ITC thermogram stated earlier. (∆S = 31.9 cal mol^−1^ K^−1^) and is advantageous for trapping of SeO_3_^2−^ by CCM/DCCM.

Studies of the H-bonding between CCM and SeO_3_^2−^ have been conducted by analyzing the second-order perturbation energy, E(2), values obtained by the NBO method^47^. The E(2) values quantify the hyperconjugative effect described by electron delocalization from the lone-pair electrons on the oxygen atom to the vacant CH sigma anti-bond: LP(O)→σ*CH. This hyperconjugative description of H-bonding^48^ accounts for the red-shifted IR stretching of the H-bonding donor (D−H)^49^, which has been described as a signature of H-bonding^50^. In the most stable CCM/SeO_3_^2−^ conformer (denoted as CS-10 in Figure S2), H-bonding can be observed for the chain and methyl hydrogen atoms (see Table 1). It is informative that the H-bonding between the methyl hydrogen(s) of CCM and the anion can be observed in other CCM/SeO_3_^2−^ adducts (B and C conformers). This type of H-bonding (between a methyl group and an anion) has also been reported for imidazole-based ionic pairs, which is well known in the field of room-temperature ionic liquids (RTILs) chemistry. In RTILs, H-bonding between an anion (PF6^−^, for example) and the alkyl hydrogen atoms detected via both NMR spin-lattice relaxation time measurements and theoretical studies has been reported^51^. According to Weinhold’s rationalization of improper H-bonding^48^, the hybrid orbital of the carbon in a CH bond that is involved in H-bonding would exhibit an increase in s-character based upon Hund’s rule. NBO analysis consistently revealed an increase in s-character (see Δs% in Table 1) going from free CCM to anion-trapping CCM. These positive values of Δs% reveal that there is H-bonding between the anion and the CCM molecule once the anion has been attracted by the electric field of the positive hydrogen atoms. This conclusion is also valid for the CS-1, CS-4, and CS-7 conformers. The steric effect exerted by the methyl group(s) is ascribed to or accompanied by H-bond formation.

**Figure 3:**
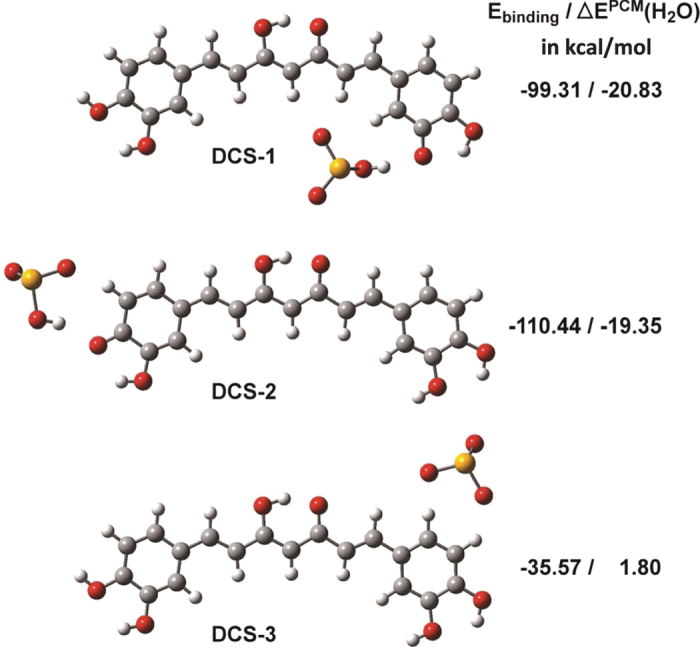
DCCM/SeO_3_^2−^ adducts with SeO_3_^2^- binded at various sites (a–c in Fig. 2a). Values of E_binding_ and ΔE^PCM^(H_2_O) in kcal/mol of DCCM/SeO_3_^2−^ adduct are shown.

To further confirm these interactions, theoretical calculations thus were performed for the DCCM/SeO_3_^2−^ adducts. The DCCM molecule has at least one non-protected protic hydrogen and is expected to be more acidic. For free DCCM, the optimized keto-enol forms (denoted as E-1~4, FI-1~4, FII-1~4, and G-1~4) and the di-keto forms (denoted as HI-1~3, HII-1~4, and HIII-1~3) are given in Figure S4 and Table S4. An analysis of these results reveals that the keto-enol conformers are lower in energy than the di-keto conformers, and all the keto-enol conformers are virtually identical in energy. Conformer G-2 is the most stable. The possible G-2/SeO_3_^2−^ adducts and the values of E_binding_ in Fig. 3 and Table S5, respectively. The binding of selenite ions lead to two major conformers of DCCM. Figure S3b has the EPPs of DCCM (E-1, FI-1, FII-1, and G-1). The CCM/SeO_3_^2−^ adduct (see Figure S2) can be described microscopically as CS-1, CS-4, CS-7, and CS-10, in which SeO_3_^2−^ is trapped at “site a” by H-bonding. The phenolic hydrogen is intramolecularly H-bonded with the neighboring methoxyl oxygen and is therefore less able to bind the anion than the methoxyl group. For DCCM, one of the phenolic hydrogens can be transferred to the anion. This explains the possible DCCM/SeO_3_^2−^ adduct shown in Fig. 3. It is also evident that anions can be trapped by both DCCM conformers, as depicted for DCS-1 and DCS-2, even if exist in water solvent (see ΔE^PCM^ in Table S5).

**Figure 4:**
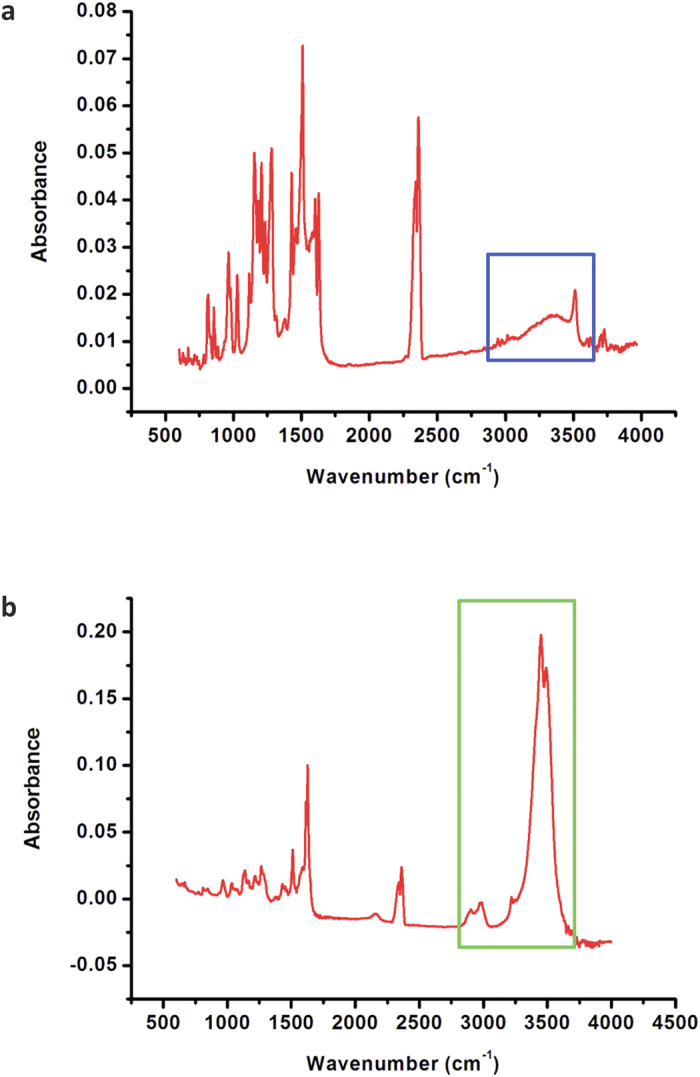
Solid-state FT-IR spectra for (**a**) CCM and (**b**) CCM/SeO_3_^2−^. The H-bonding signals are highlighted by boxes (CCM: blue box; CCM/Selenite: green box; details are listed in Table S6).

The anion-trapping properties of CCM have been examined with IR spectrometry, and numerous efforts have been made to rationalize (1) the increase in the IR intensity of the stretching band of the H-bonding donor (D−H stretching)^52^,^53^,^54^ and (2) the unusual blue shift of the D−H stretching (termed improper H-bonding)^46^. The solid-state IR spectra for CCM and CCM/SeO_3_^2−^ are shown in Fig. 4, respectively. The spectral assignments are summarized in Table S5. The increases in intensity and the low-frequency shifts (see Table S5,6 or Fig. 4) of the phenolic O−H stretching, which occurs at 2940–3020 cm^−1^ for free CCM and 2940–2990 cm^−1^ for CCM/SeO_3_^2−^, indicate the formation of H-bonds between this motif of CCM and selenite^52^,^53^,^54^. The red-shifts in the C−H stretching bands of the methyl group, from 2940–3020 cm^−1^ in free CCM to 2940–2990 cm^−1^ in CCM/SeO_3_^2−^, can be explained by H-bond formation between the phenolic methyl group and selenite. This explanation is also found for the O−H stretching bands. Compared to the methoxyl oxygen atom, the negatively charged oxygen atoms of selenite serve as better H-bond acceptors. In the close-contacting state, the intra-molecular H-bonding in free CCM is replaced by the H-bonding described by the OH…O moiety (selenite). This H-bonding is confirmed even more convincingly by the changes in intensity of the methyl C–H and phenyl O–H signals (see Fig. 4). The pronounced decrease in intensity observed for the absorption peaks ranging from 1600 to 800 cm^−1^ (see assignments in Table S5 and Figure S6) also supply evidence from the IR analysis in support of CCM/anion binding, as predicted by the DFT calculations described earlier^20^. In Figure S5, the IR spectra of CCM (C form) and the CCM/SeO_3_^2−^ complex CS-10 were also calculated, and the results, which indicate an increase in intensity for the C−H stretching (labelled ν1) and a decrease in intensity for the C = C stretching and C−H bending (labelled ν2), are similar to the experimental observations. From examination of the IR studies repeated for DCCM, it was observed that the O−H stretching bands virtually disappeared. This is very instructive because the proton of DCCM transfers to selenite, as predicted by the DFT calculations (see Figure S7), implying that an ionic bond is formed. The results of these IR experiments reveal that H-bonding plays a major role in the binding or trapping of selenite at site a and that this H-bonding is enhanced by the two methyl groups, as depicted in Fig. 2 and quantified in Table 1. For DCCM, the major driving force of the interaction is the formation of an acid-base adduct with selenite.

**Figure 5:**
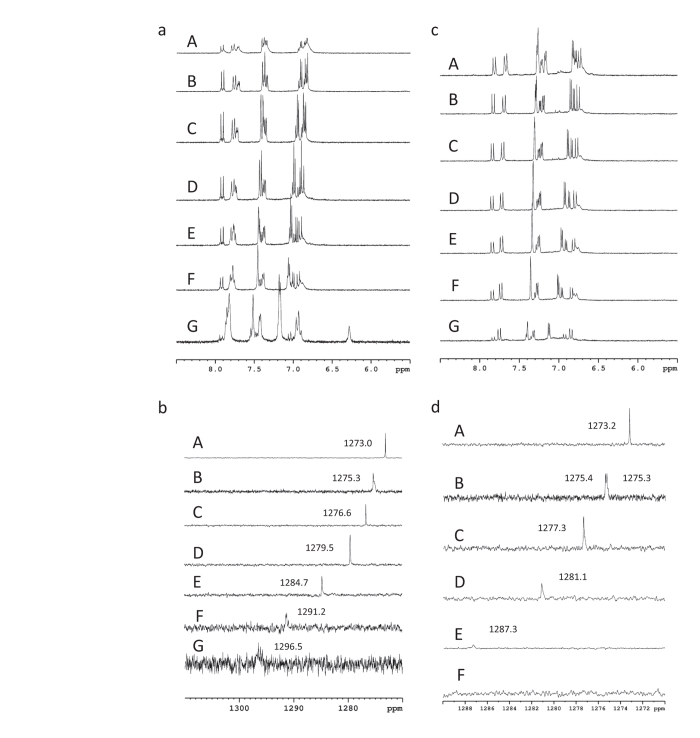
NMR spectra. (**a**) ^1^H-NMR spectra for CCM (6 mM) with various ratios of DCCM/SeO_3_^2−^ in ACN-d3/ D_2_O (3/7). CCM/SeO_3_^2−^: (**A**) 1/32, (**B**) 1/16, (**C**) 1/8, (**D**) 1/4, (**E**) 1/2, (**F**) 1/1, and (**G**) 1/0. (**b**) ^77^Se-NMR spectra for CCM (6 mM) with various ratios of CCM/SeO_3_^2−^ in ACN-d3/ D_2_O (3/7). SeO_3_^2−^/CCM: (**A**) 32/0, (**B**) 32/1, (**C**) 32/2, (**D**) 32/4, (**E**) 32/8, (**F**) 32/16, and (**G**) 32/32. (**c**) ^1^H-NMR spectra for DCCM (6 mM) with various ratios of DCCM/SeO_3_^2−^ in methanol/ACN-d3/D_2_O (0.5:3:6.5). DCCM/SeO_3_^2−^: (**A**) 1/32, (**B**) 1/16, (**C**) 1/8, (**D**) 1/4, (**E**) 1/2, (**F**) 1/1, and (**G**) 1/0. (**d**) ^77^Se-NMR spectra for DCCM (6 mM) with various ratios of DCCM/SeO_3_^2−^ in methanol/ACN-d3/D_2_O (0.5:3:6.5). SeO_3_^2−^/DCCM: (**A**) 32/0, (**B**) 32/1, (**C**) 32/2, (**D**) 32/4, (**E**) 32/8, and (**F**) 32/16. The full details of the chemical shifts are listed in Tables S7 and S8, and the spectra are shown in Figure S8 and S9.

Concentration-dependent ^1^H- and ^77^Se-NMR spectra recorded for CCM/SeO_3_^2−^ mixtures are shown in Fig. 5a. Compared to reported ^1^H-NMR recorded for the pure (selenite free) CCM solution^39^,^55^, the additional resonance peaks in Fig. 5a reveals co-existence of a second conformer in the presence of acetonitrile as observed in the present work. This observation resembling the Ga(III)-CCM ^1^H-NMR spectra^55^ was ascribed to the appearance of diketone structure (see page 66 of reference 55). Thus, we conclude that acetonitrile-associated CCM in di-ketone conformation, denoted by MeCN…CCM (di-ketone), was responsible for the additional resonance signals found in Fig. 5a. The latter is proposed based on the H-bonding between acetonitrile^56^ and the enolic hydrogen. For the DCCM analog shown in Fig. 5c, one seen that the MeCN…DCCM (di-ketone) complex appears even pronounced, which is explained by the more acidic nature of DCCM as compared to CCM. In simple words, existence of basic solution (acetonitrile) would stabilize the di-ketone conformer in the keto-enol and di-keto tautomeric equilibrium. In the presence of SeO_3_^2−^, the uniformly up-field shifts and decreasing in intensity of all proton resonance signals of free CCM as increasing selenite. On the other hand, the resonance signals of MeCN…CCM show increasing intensities (also show up-field shifts) as the contents of SeO_3_^2−^ increase. The concentration-dependent proton and ^77^Se-NMR spectra (available in Fig. 5b,d) show constantly down-field shifts with increasing contents of SeO_3_^2−^. Those NMR data in a whole can be explained by interaction between CCM and selenite as the argument employed to account for CD-CCM complexation in the study of refinement of anti-cancer activity of CCM-derivatives^39^. The interactions derived from NMR data are most plausibly ascribed to proton transfer from CCM to SeO_3_^2−^ in solution states, which can be described by CCM + SeO_3_^2−^ ⇌ CCM^−1^ + HSeO_3_^−^. These explanations are plausible considering the basic nature of SeO_3_^2−^ and the acidic nature of CCM. The fast proton migration give the fluctuational nature of either  ^1^H- or ^77^Se-NMR spectra as found HOSEY NMR data found for ionic liquids^57^,^58^. More significantly, the up-field shifts of the methyl hydrogens can be understood through the above-stated proton transfer concept because H-bonding with SeO_3_^2−^ would lead to down-field shifts in the resonance signals of the methyl hydrogens. The Lewis base nature of SeO_3_^2−^ must be considered because it is a weaker acid than acetic acid, while CCM has been recognized as an acid. H-bonding donors derived from methyl groups have been observed previously by Carper and colleagues through theoretical studies of a BMI/PF6 ionic liquid.^51^

Similarly, one of the methyl groups in the CCM/SeO_3_^2−^ complex CS-10 can interact with SeO_3_^2−^ (see Figure S2) through H-bonding, as indicated by the hydrogen-oxygen inter-nuclear distances and the E(2) analysis described earlier. It is noteworthy that, unlike traditional H-bonding, some of the methyl C–H bond lengths involved in H-bonding with the anion might be shorter than these lengths in free CCM (see Table 1). The NBO analysis of those CH bond orbitals revealed that the s-characters of the hybrid orbitals of the carbon atoms involved in forming CH bonds increase in the presence of SeO_3_^2−^, an observation that is consistent with the improper H-bonding rationalized by the rehybridization concept proposed recently by Weinhold^48^. The enolic proton is more acidic than the phenolic protons based upon the reported pKa values^59^. Furthermore, the H4 proton is mobile^59^ and might be considered a proton donor in the presence of SeO_3_^2−^. The resonance signal disappeared in the presence of SeO_3_^2−^, which is indicative of an acid-base interaction between CCM and SeO_3_^2−^. Similar NMR data obtained for DCCM and DCCM/selenite (see Fig. 5c,d) are also consistent with this conclusion. The MeCN…DCCM association is more pronounced than that of MeCN…CCM, and this result is manifested by the more pronounced additional signals in neat CCM or DCCM. This can be explained by the more acidic nature of DCCM. In other words, the NMR and IR data are consistent with the nature of the selenite binding. Through the ITC results, CCM and selenite (Fig. 1b) have an endothermic titration curve with a stoichiometry of n = 1.22, whereas DCCM and selenite (Fig. 1c) have an endothermic titration curve with a stoichiometry of n = 1.90. These results can be understood based upon the DFT results given in Figures S2,3. The DCCM molecule can accommodate selenite through two sites via acid-base interactions. For the CCM molecule, the two central oxygen atoms are accompanied by a negatively charged potential, as displayed by the EPPs in Figure S3, which excludes the approach of the selenite anion.

Our results show that 30 μM DCCM resulted in significant inhibition on turbidity formation, whereas 100 μM CCM was required to obtain inhibition. The di-ketone CCM analogues were responsible for the biomedical effect, as indicated by this study. We therefore propose equilibrium of keto-enol ⇌ di-ketone CCM analogue tautomerization as an explanation for the reported biomedical activities. The complex formation of selenite with derivatives of CCM resulted in extenuating the toxicity of selenite as to prevent selenite induced protein aggregation. It is also reported that selenite would interact with organic fractions of natural grassland soil^60^, hence, selenite sorption by organic compounds may be a common phenomenon. The other organic compounds with these chemical properties can be expected.

In conclusion, our results provide an explanation how CCM and DCCM prevent selenite induced lens protein aggregation, which may lead to different strategies for anti-cataract drug design. Whether overdosing CCM/DCCM results in selenite deficiency *in vivo* due to CCM (DCCM)-selenite complexation is warrant further investigation.

## Methods

### *In vitro* Lens Crystallin Turbidity Assays

Porcine lenses were decapsulated and homogenized in buffer containing 50 mM Tris-HCl, 0.1 M NaCl, 5 mM EDTA, 0.01% β-mercaptoethanol, and 0.02% sodium azide, pH 8.0. After centrifugation at 16,060 g for 30 min, the supernatant was collected, and the protein concentration was determined according to the Bradford method (BioRad Laboratories, USA). The following crystallin turbidity assays were used for CCM: an *in vitro* anti-cataract screening system resulting from excess sodium selenite and lens protein turbidity was formed. Lens proteins with buffer only were used as normals. Crystallins with selenite (Na_2_SeO_3_, 10 mM) ions were used as controls. Buffers with or without CCM were used as blanks. Samples were incubated at 37 °C for 4 days. The turbidity was measured at 630 nm for the selenite induced turbidity experiments. The same method was used for the didemethylated CCM (DCCM).

### FT-IR spectroscopy of CCM and the CCM/SeO_3_^2−^ Mixtures

For the CCM/ionic salt preparation, CCM (20 μM) was mixed with ionic salt (200 μM) in a buffer containing ACN (ACN/H_2_O = 1/100 by volume). The mixture was then freeze-dried under vacuum. The IR spectra were measured with a Bruker Tensor27 FT-IR device (Bruker, USA).

### Isothermal Titration Calorimetry of the CCM/SeO_3_^2−^ Mixtures

The binding thermodynamics of the selenite and CCM was measured with an iTC200 system (GE, MicroCal, USA). The injector syringe was programmed to titrate the CCM solution into the cell with 1.9 μl per injection at 180 s intervals. The stirring rate of the injector was 1,000 rpm. In control experiments, the CCM solution was injected under the same conditions into a propanol/water solution. The raw calorimetry data were collected and analyzed using Origin version 7.0 data analysis software (GE, MicroCal, USA). The binding isotherms were fitted to the one-set of site binding model to obtain values for the binding ratio (n), the changes in enthalpy (ΔH) and entropy (ΔS) of binding, and the binding constant (KD). The Gibbs free energy of binding, ΔG, was also calculated using the following equation: ΔG = –RTln(1/KD) = ΔH – TΔS. The thermograms of CCM and 1,7-bis(3,4-dihydroxyphenyl)-1,6-heptadiene-3,5-dione were performed by separately titrating CCM (0.4 mM) and 1,7-bis(3,4-dihydroxyphenyl)-1,6-heptadiene-3,5-dione (0.4 mM) with selenite (8 mM) in a propanol/water (1/1) solution.

### ^1^ H-NMR Spectra

NMR experiments were performed with a Bruker Avance NMR spectrometer (400 and 500 MHz) at 300 K. The chemical shifts in the ^1^H-NMR spectra were reported relative to D_2_O (δH, 4.67 ppm).

### ^77^Se-NMR Titration Studies of the CCM/Selenite Complex

NMR experiments were performed with a Bruker Avance NMR spectrometer (400 MHz) at 300 K. The ^77^Se-NMR spectra were reported relative to external diphenyl diselenide (PhSeSePh, 460 ppm).

### Didemethylation of CCM

The DCCM, 1,7-bis(3,4-dihydroxyphenyl)-1,6-heptadiene- 3,5-dione was synthesized as reported previously^61^. Anhydrous AlCl_3_ (2.67 g, 20 mmol) was suspended in a solution of CCM (2.2 g, 6 mmol) in CH_2_Cl_2_ (100 mL) in an apparatus protected from atmospheric moisture. Pyridine (6.4 mL, 80 mmol) was added dropwise with stirring. The mixture was refluxed for 24 h, cooled with ice, and acidified with dilute HCl(aq). The aqueous phase was subjected to several EtOAc extractions. The combined organic layers were separated and evaporated to dryness. The residue was purified on a silica gel column by eluting with CH_2_Cl_2_-MeOH to give 0.88 g (43%) 1,7-bis(3,4-dihydroxyphenyl)-1,6-heptadiene-3,5-dione.

### Computational Methods

The hybrid functional M06-2X^62^ of Truhlar and Zhao was used for all calculations. The geometric optimizations and frequency calculations (IR report) were carried out by 6–31 + G(d) basis set, and single point energy calculations in vacuum or solvent were done by 6–311 + G(2 df,2p) in order to obtain more accurate energies and electronic properties (included electronic potential plots (EPP) and natural bond orbital (NBO) analysis). 6–31 G(d) only was used to compare basis set effect, and correlated discussion was putted into support materials (the part of “BSSE and selection of basis set”). The three difference basis set predicted similar result for the relative energies of CCM monomers, but the binding energies (E_binding_) of CCM/SeO_3_^2−^ adduct showed large difference. Because the basis set extension accompanies with reducing extant of basis set superposition error BSSE, especially the adding diffuse functions is definitely beneficial effect in anion-neutral system^63^,^64^,^65^. The BSSE calculated by counterpoise method^66^,^67^ can demonstrate this phenomenon (see “BSSE and selection of basis set” of support materials). The E_binding_ of the CCM/SeO_3_^2−^ and DCCM/SeO_3_^2−^ adducts are calculated according to the following relationship:


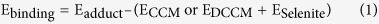


Solvent effect included water and ethanol was calculated by using CPCM solvation model^68^,^69^. The corrected energy and E_binding_ are labeled as E^PCM^(solvent) and ΔE^PCM^(solvent), respectively. The bond orbital natures and E(2) values were obtained using NBO version 3.1^47^,^70^,^71^,^72^,^73^,^74^,^75^, and all the calculations were performed with the Gaussian 09 package^76^.

## Additional Information

**How to cite this article**: Liao, J.-H. *et al.* The Comparative Studies of Binding Activity of Curcumin and Didemethylated Curcumin with Selenite: Hydrogen Bonding vs Acid-Base Interactions. *Sci. Rep.* **5**, 17614; doi: 10.1038/srep17614 (2015).

## Supplementary Material

Supporting Material

## Figures and Tables

**Table 1:** Values of E(2)^a^, changes in s-character (Δs%), and changes in the CH bond lengths (Δr_CH_) going from free CCM to the CS-10 CCM/SeO_3_^2−^ adduct, and CH…O distances (r_CH…O_) in the static CS-10 adduct.

	total E(2)^a^ of LP(O) → σ*_CH_ (kcal/mol)	Δs%	Δr_CH_ (Å)	r_CH…O_(Å)
vinyl and phenolic CH anti-bond				
σ*_C2-H_	10.24	1.28%	0.009	1.967
σ*_C4-H_	6.54	1.71%	0.009	2.176
σ*_C6-H_	6.74	1.32%	0.004	1.990
σ*_C9-H_	9.71	3.04%	0.012	1.999
σ*_C15-H_	8.72	2.91%	0.011	1.975
Methoxyl CH anti-bond				
σ*_C10-Ha_	0.36	1.07%	−0.004	2.549
σ*_C10-Hb_	0.72	0.98%	−0.005	2.509
σ*_C16-Ha_	0.12	0.61%	−0.003	2.692
σ*_C16-Hb_	0.99	1.10%	−0.004	2.448

^a^Value of E(2) represent the electron delocalization energy associated with the donor-acceptor hyperconjugative interactions expressed by LP(O) → σ*_CH_.
