# Supplementary material for: The Comparative Studies of Binding Activity of Curcumin and Didemethylated Curcumin with Selenite: Hydrogen Bonding vs Acid-Base Interactions
Source: Sci Rep. 2015 Dec 4;5:17614. doi: 10.1038/srep17614 (PMC4669449; doi:10.1038/srep17614)
Supplement: Supporting Material [file srep17614-s1.pdf]

Supplementary Data
for

The Comparative Studies of Binding Activity of Curcumin and Didemethylated Curcumin with Selenite: Hydrogen Bonding vs Acid-Base Interactions

Jiahn-Haur Liao[a, ‡], Tzu-Hua Wu[b, ‡], Ming-Yi Chen[c], Wei-Ting Chen[d], Shou-Yun Lu[d], Yi-Hsuan Wang [d], Shao-Pin Wang[d], Yen-Min Hsu[e], Yi-Shiang Huang[a, b], Zih-You Huang[e], Yu-Ching Lin[a], Ching-Ming Chang[e], Fu-Yung Huang[d,*], Shih-Hsiung Wu[a, e,]

[a]Institute of Biological Chemistry, Academia Sinica, Taipei 11529, Taiwan; [b]Department of Clinical Pharmacy, School of Pharmacy, College of Pharmacy, Taipei Medical University, Taipei 110, Taiwan; [c]General Education Center, National Taipei University of Nursing and Health Sciences, Taipei, Taiwan; [d]Department of Chemistry, National Cheng Kung University, Tainan 701 Taiwan; [e]Institute of Biochemical Sciences, National Taiwan University, Taipei 10617, Taiwan

KEYWORDS: curcumin • didemethylated curcumin • selenite • cataract • hydrogen bonding

‡ Equal contribution to this work.

Corresponding Author
* To whom correspondence should be addressed: Shih-Hsiung Wu, PhD, Tel: 8862 2785 5696, Ext. 7101; Fax: 8862 2653 9142, E-mail: shwu@gate.sinica.edu.tw; Fu-Yung Huang, PhD, Tel.: 8866 2757 575 ext. 65353, Fax: 8866 2740 552, E-mail: fhuang@mail.ncku.edu.tw

Tables

Table S1. The electronic energies in vacuum (E), in two solvents (E^{PCM}) obtained by M06-2X/6-311+G(2df,2p)//M06-2X/6-31+G(d)[a]. The results in vacuum carried out using M06-2X with 6-31G(d) and 6-31+G(d) also are shown. See Figure S1 for denoting various conformers.

**basis set**	**condition**	**E or E^{PCM} (hartree)**	**ΔE or ΔE^{PCM} (kcal/mol)**					
		A	BI	BII	C	DI	DII	DIII
6-31G(d)	vacuum	-1263.049844	-0.12	-0.14	-0.17	3.30	2.84	2.19
6-31+G(d)	vacuum	-1263.092122	-0.12	-0.14	-0.20	3.70	3.15	2.41
6-311+G(2df,2p)	vacuum	-1263.506285	-0.15	-0.16	-0.26	6.95	6.42	5.69
	H_2O	-1263.524463	-0.39	-0.36	-0.78	5.54	5.17	4.71
	CH_3OH	-1263.523887	-0.37	-0.35	-0.75	5.58	5.21	4.73

[a] Energies of conformers A are presented in Hartree; while corresponding values of conformers B-D are given in kcal/mol compared to conformers A.

Table S2. Values of $E_{binding}$[a] and corrected BSSE-corrected $E_{binding}$ ($E_{binding}$ / $E_{binding(correction)}$) obtained for conformers CS-1, CS-4, CS-7, CS-10. M06-2X calculations with the 6-31G(d), 6-31+G(d) and 6-311+G(2df,2p)[a] basis set. set. See Figure S2 for denoting conformers.

	**$E_{binding}$ / $E_{binding(correction)}$ (kcal/mol)**		
	6-31G(d)	6-31+G(d)	6-311+G(2df,2p)[a]
CS-1	-83.06 / -53.50	-64.51 / -52.53	-55.48 / -[b]
CS-4	-98.87 / -69.38	-80.49 / -[b,c]	-71.03 / -[b]
CS-7	-100.64 / -66.10	-79.44 / -66.79	-70.09 / -[b]
CS-10	-116.95 / -80.13	-93.21 / -80.27	-83.77 / -[b]

[a] M06-2X/6-311+G(2df,2p)//M06-2X/6-31+G(d) is used. [b] Self-consistent field performed on SeO_3^{2-} fragment in adduct basis did not converge. [c] All trials from quadratically convergent SCF procedures failed.

Table S3. Binding energies obtained for CCM-SeO_3^{2-} adducts in vacuum (ΔE) and two solvents (ΔE^{PCM}) obtained by M06-2X/6-311+G(2df,2p)//M06-2X/6-31+G(d) calculations. Four keto-enol conformers are shown here; C is negligibly lower (by less than 1.0 kcal/mol) in energy than the other three conformers, which predicts that all four of the conformers should be virtually identically populated in a solution state. See Figure S2 for denoting various conformers.

	ΔE ($E_{binding}$)[a] (kcal/mol)	ΔE^{PCM}(H_2O)[a] (kcal/mol)	ΔE^{PCM}(CH_3OH)[a] (kcal/mol)
A conformer			
CS-1	-55.22	1.03	-0.11
CS-2	-51.27	4.16	2.97
CS-3	-49.05	3.31	2.19
BI conformer			
CS-4	-70.92	2.26	0.52
CS-5	-51.77	3.92	2.72
CS-6	-35.37	2.11	1.64
BII conformer			
CS-7	-70.00	2.67	0.95
CS-8	-36.97	2.78	2.18
CS-9	-49.51	3.06	1.93
C conformer			
CS-10	-83.77	1.63	-0.55
CS-11	-37.45	2.51	1.91
CS-12	-35.87	1.82	1.36

[a] The energy of conformer C was used for monomer.

Table S4. The electronic energies in vavuum (E) and two solvents (E^{PCM}) of DCCM were obtained using M06-2X/6-311+G(2df,2p)//M06-2X/6-31+G(d). See Figure S3 for denoting various conformers.

	E (hartree)	$E^{PCM}(H_2O)$ (hartree)	$E^{PCM}(CH_3OH)$ (hartree)
keto-enol form			
EI-1	-1184.921107	-1184.943200	-1184.942502
EI-2	-1184.920446	-1184.943025	-1184.942301
EI-3	-1184.920333	-1184.943034	-1184.942303
EI-4	-1184.919587	-1184.942857	-1184.942094
FI-1	-1184.921175	-1184.943667	-1184.942947
FI-2	-1184.920423	-1184.943468	-1184.942718
FI-3	-1184.921060	-1184.943520	-1184.942802
FI-4	-1184.920403	-1184.943360	-1184.942616
FII-1	-1184.921204	-1184.943640	-1184.942924
FII-2	-1184.921153	-1184.943517	-1184.942804
FII-3	-1184.920341	-1184.943468	-1184.942713
FII-4	-1184.920373	-1184.943349	-1184.942603
G-1	-1184.921175	-1184.944130	-1184.943385
G-2	-1184.921293	-1184.944032	-1184.943300
G-3	-1184.921229	-1184.944008	-1184.943274
G-4	-1184.921127	-1184.943902	-1184.943167
di-keto form			
HI-1	-1184.909903	-1184.934309	-1184.933544
HI-2	-1184.909318	-1184.934105	-1184.933320
HI-3	-1184.908907	-1184.933903	-1184.933108
HII-1	-1184.910611	-1184.934715	-1184.933961
HII-2	-1184.909696	-1184.934576	-1184.933787
HII-3	-1184.910228	-1184.934499	-1184.933737
HII-4	-1184.909113	-1184.934404	-1184.933594
HIII-1	-1184.911499	-1184.935236	-1184.934499
HIII-2	-1184.910469	-1184.935062	-1184.934285
HIII-3	-1184.909454	-1184.934939	-1184.934119

Table S5. Binding energies obtained for DCCM(G-1)-SeO_3^{2-} adducts in vacuum (ΔE) and two solvents (ΔE^{PCM}) obtained by M06-2X/6-311+G(2df,2p)//M06-2X/6-31+G(d) calculations. See Figure 3 for denoting various conformers.

	ΔE ($E_{binding}$) (kcal/mol)	ΔE^{PCM}(H_2O) (kcal/mol)	ΔE^{PCM}(CH_3OH) (kcal/mol)
DCS-1	-99.31	-20.83	-22.83
DCS-2	-110.44	-19.35	-21.73
DCS-3	-35.57	1.80	1.32

Table S6. Comparison of FT-IR vibrational frequencies recorded for free CCM and CCM/SeO_3^{2-} adduct between those predicted by M06-2X/6-31+G(d) calculations, given in parenthesis carried out on conformers of C and CS-10.

CCM	CCM/SeO_3^{2-}	Assignments from Yallapua [a]	Assignments in this work	Reference spectroscopy book [b]
3512 (3772.69, 3772.97)	3489, 3449 (3746.23, 3735.29)	3510, phenolic OH stretching	phenolic OH stretching	phenolic OH stretching, 3610
3015, 2973, 2945 (3203.08, 3202.50, 3136.59, 3136.32, 3068.28, 3068.19)	2987, 2901 (3226.01, 3210.74, 3205.29, 3187.57, 3118.27, 3100.92)		1. CH3 stretching (symm/asymm) 2. CH stretching for RCH=CHR	1. R-O-CH3 stretching: 2992-2955 (asymm), 2897-2865 (symm) 2. CH stretching in RCH=CHR, 3020
1627 (1750.40, 1725.89)	1627 (1757.09, 1739.60)		C=C stretching in C=CH-CO	C=C stretching in C=CH-CO, 1660-1580
1602 (1697.38, 1691.56, 1684.11, 1680.81)	no (1697.47,1694.89, 1689.15, 1678.61, 1677.40)	1605, benzene ring stretching	benzene ring stretching	C=C stretching in skeletal phenyl, 1600 (p. 79)
1508 (1668.07)	1510 (1649.93)	1502, C=O and C=C stretching	C=O stretching, and C=C stretching for skeletal phenyl	1. C=C stretching in skeletal phenyl, 1500 2. C=O stretching in beta-di-ketone, 1640-1535
1458 (1534.37, 1533.95, 1521.98, 1521.72)	no (1517.83, 1513.66, 1511.78, 1502.69)		CH3 bending of R-O-CH3	CH3 bending of R-O-CH3: 1. symm, 1455-1430. 2. on symm, 1470-1440
1429 (1498.60)	1429 (1454.60, 1467.18)	1435, olefinic C-H bending	=CH2 scissoring	=CH2 scissoring, 1420
1281 (1210.92, 1209.98)	1216 (1248.15, 1193.91)	1285, aromatic C-O stretching	aromatic C-O-C stretching (asymm)	aralkyl (asymm), 1270-1230
1027 (1100.55, 1100.11)	1035 (989.06, 982.68)	1027, C-O-C stretching	aromatic C-O-C stretching (symm)	C-O-C stretching (symm), 1120-1030
856 (849.99, 847.41)	no (831.42, 822.10, 819.17, 813.94)	840, C-O-C stretching	C-H out-of-plane bending (2-adj H-atoms)	C-H out-of-plane bending (2-adj H-atoms), 860-800

[a] M. M. Yallapua, M. Jaggi, S. C. Chauhana, *Colloids Surf., B* **2010**, *79*, 113-125.

[b] J. Mohan, *Organic Spectroscopy: Principles and Applications*, Narosa Publishing House, New Delhi, **2001.**

Table S7. ^{1}H- and Se^{77}-NMR chemical shifts (ppm) of curcumin and the curcumin/SeO_3^{2-} complex in ACN-*d3*/D_2O.[a]

	Proton							Se^{77}
Ratio Se/Cur	**H1/7**	**H2/6**	**H4**	**H9/15**	**H12/18**	**H13/19**	**-OCH_3**	Se^{77}
32:0	-	-	-	-	-	-	-	1273.0
32:1	7.76	6.82	-	7.38	6.90	7.34	4.03	1275.3
	7.80	6.86		7.41	6.91	7.36	4.08	
	7.90	6.87			6.94	7.38		
	7.94	6.91			6.95	7.40		
16:1	7.74	6.82	-	7.36	6.89	7.33	4.00	1276.6
	7.77	6.85		7.39	6.90	7.35	4.05	
	7.89	6.83			6.91	7.36		
	7.92	6.86			6.93	7.38		
8:1	7.75	6.83	-	7.39	6.93	7.34	4.02	1279.5
	7.78	6.86		7.41	6.95	7.36	4.07	
	7.90	6.87			6.95	7.37		
	7.93	6.90			6.97	7.39		
4:1	7.76	6.85	-	7.40	6.98	7.35	4.03	1284.7
	7.79	6.88		7.43	7.00	7.37	4.08	
	7.89	6.89			7.00	7.38		
	7.93	6.92			7.01	7.40		
2:1	7.77	6.87	-	7.42	7.02	7.37	4.05	1291.2
	7.80	6.90		7.44	7.03	7.39	4.09	
	7.90	6.92			7.03	7.40		
	7.93	6.95			7.05	7.42		
1:1	7.81	6.91	-	7.45	7.04	7.37	4.07	1296.5
	7.85	6.95			7.05	7.39	4.10	
	7.90	6.99			7.06	7.41		
	7.94	7.03			7.07	7.43		
0:1	7.85	6.96	-	7.85	7.16	7.42	4.14	
	7.82	6.93			7.18	7.43		

[a] Curcumin (6 mM) in ACN-*d3*/D_2O(3:7). The complexation of curcumin/SeO_3^{2-} and curcumin(6 mM) with various ratios of curcumin/Na_2SeO_3 (0:1 to 32:0).

Table S8. ^{1}H- and Se^{77}-NMR chemical shifts (ppm) of didemethylated curcumin and didemethylated curcumin /SeO_3^{2-} complex in ACN-d_3/D_2O.[a]

	Proton							Se^{77}
	**H1/7**	**H2/6**	**H4**	**H9/15**	**H12/18**	**H13/19**	**-OH**	Se^{77}
Ratio Se/Cur								
32:0	-	-	-	-	-	-	-	1273.166
32:1	7.656	6.722	-	7.260	6.779	7.160	-	1275.277
	7.687	6.753		7.264	6.795	7.164		1275.408
	7.800			7.276	6.813	7.180		
	7.831			7.280	6.830	7.176		
						7.208		
						7.212		
						7.224		
						7.228		
16:1	7.675	6.740	-	7.281	6.801	7.183	-	1277.338
	7.707	6.771		7.285	6.818	7.187		
	7.813			7.292	6.842	7.199		
	7.844			7.297	6.858	7.203		
						7.225		
						7.229		
						7.242		
						7.246		
8:1	7.692	6.759	-	7.302	6.829	7.205	-	1281.094
	7.724	6.791		7.307	6.845	7.209		
	7.822			7.308	6.876	7.222		
	7.854			7.313	6.892	7.226		
						7.241		
						7.245		
						7.258		
						7.262		
4:1	7.705	6.780	-	7.323	6.862	7.227	-	-
	7.737	6.812		7.325	6.879	7.231		
	7.829				6.915	7.244		
	7.860				6.932	7.247		
						7.257		
						7.262		
						7.274		

						7.278		
2:1	7.708	6.755	-	7.336	6.901	7.242	-	-
	7.739	6.798			6.917	7.258		
	7.824	6.787			6.954	7.267		
	7.856	6.830			6.971	7.284		
1:1	7.717	6.776	-	7.355	6.952	7.261	-	-
	7.749	6.804			6.968	7.278		
	7.825	6.825			7.000	7.284		
	7.856	6.857			7.016	7.301		
0:1	7.735	6.832	-	7.394	7.115	7.309	-	-
	7.767	6.864		7.411	7.131	7.325		
	7.813	6.909				7.334		
	7.844	6.941				7.350		

[a] Didemethylated curcumin (6 mM) in methanol/ACN-d_3/D_2O(0.5:3:6.5). The complexation of didemethylated curcumin /SeO_3^{2-} and didemethylated curcumin (6 mM) with various ratios of didemethylated curcumin/Na_2SeO_3(0:1 to 32:0).

Figures

Figure S1. Possible conformers for a free CCM molecule obtained by M06-2X/6-31+G(d) calculations, including keto-enol form (A-C) and di-keto form (D).

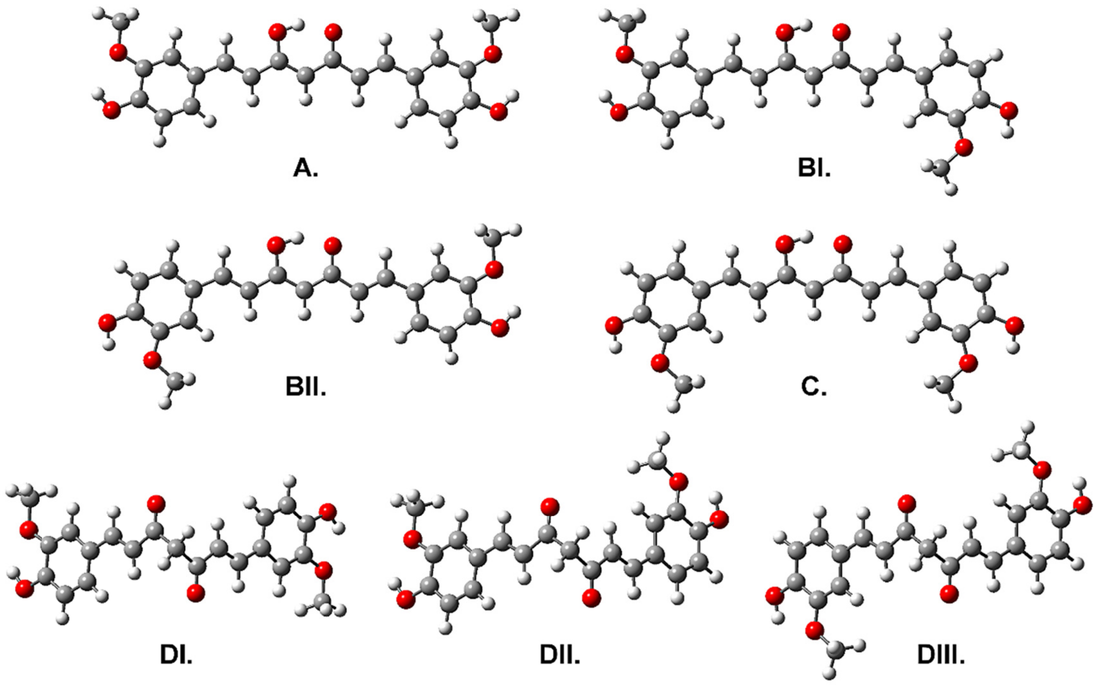

Figure S2. Possible conformers for a CCM/SeO_3^{2-} adduct (CS) obtained by M06-2X/6-31+G(d) calculations. The three sites provided by a CCM molecule to trap SeO_3^{2-} have been labeled as a, b, c as presented in Figure 2 in the content.

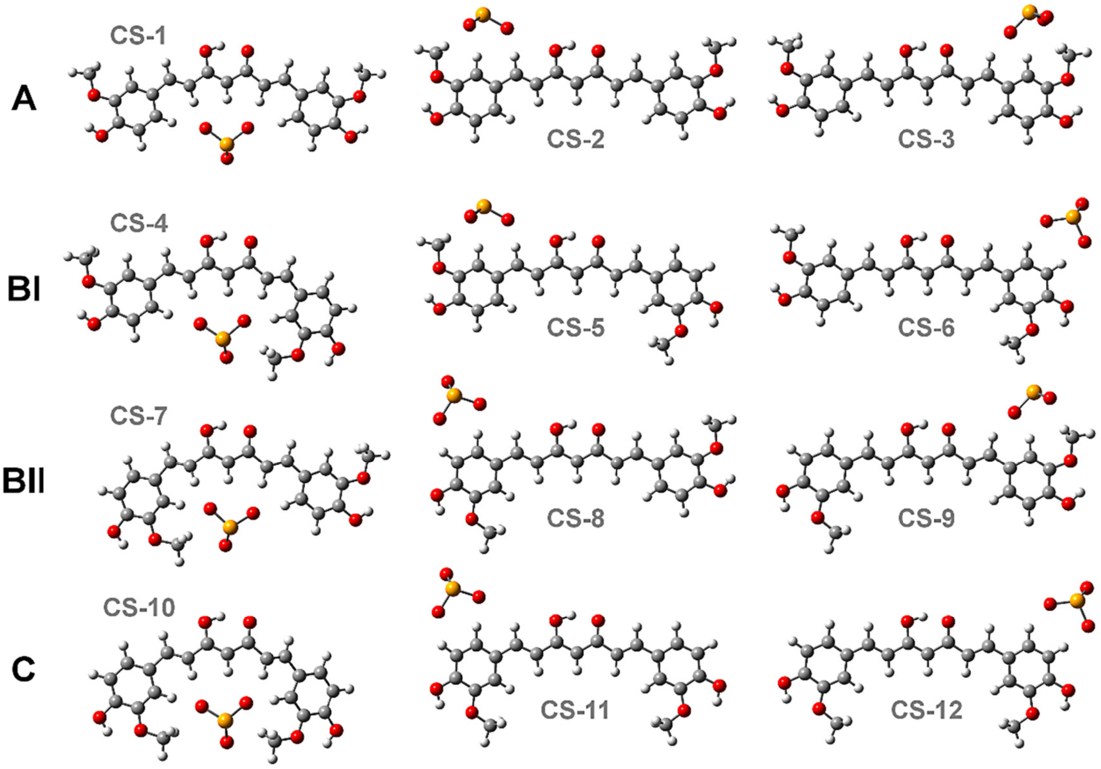

Figure S3. Electronic potential plots (EPP) obtained by M06-2X/6-311+G(2df,2p) //M06-2X/6-31+G(d) calculations of (a) four keto-enol conformers A, BI, BII, and C shown in Figure S1 and (b) four DCCM analogues denoted by E-1, **F**I-1, FII-1, and G-1 shown in the subsequent Figure. Isovalues in each EPP range from 0.01 (outer) to 0.1 (inner) with an increment of 0.01.

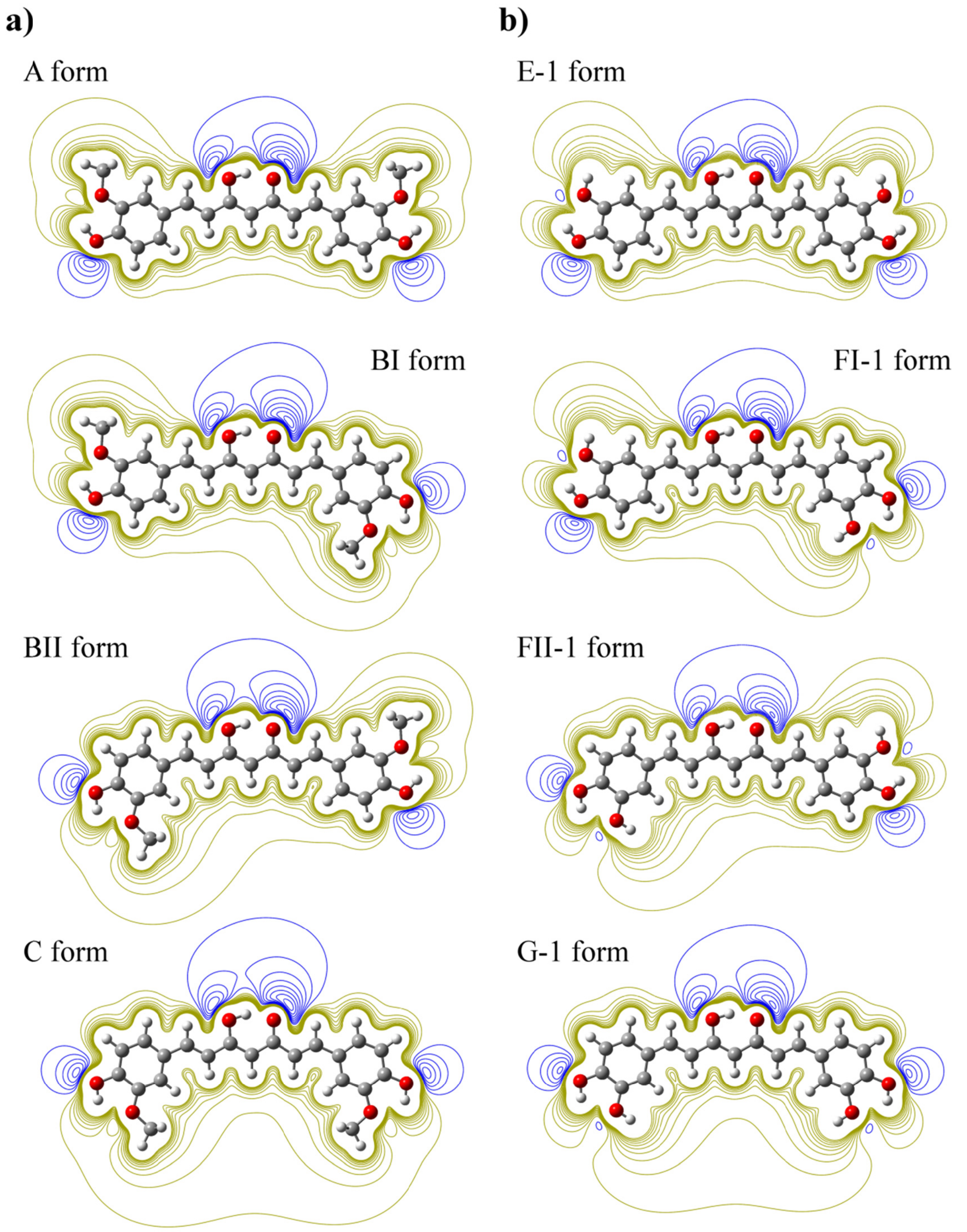

Figure S4. Possible conformers for a free DCCM molecule obtained by M06-2X/6-31+G(d) calculations, including keto-enol form (E-G) and di-keto form (H)

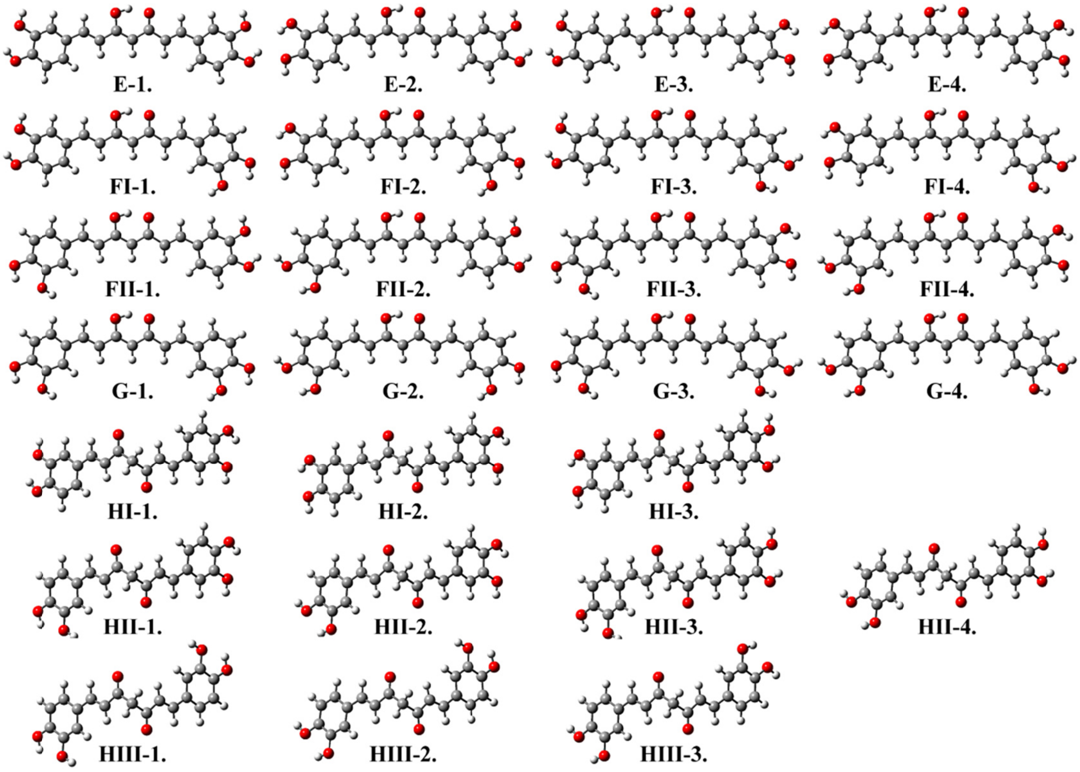

Figure S5. The predicted IR spectra for C-conformer in Figure S1 of CCM (band profiles in green) and CS-10 of the CCM/SeO_3^{2-} adduct (in black) by M06-2X/6-31+G(d) calculations. The *v*1 region mainly represents C-H bond bending and the adjacent region mainly represents C=C bond stretching. The *v*2 region mainly represents C-H stretching, which include methyl, phenyl and olefinic, overlapped with phenolic O-H stretching.

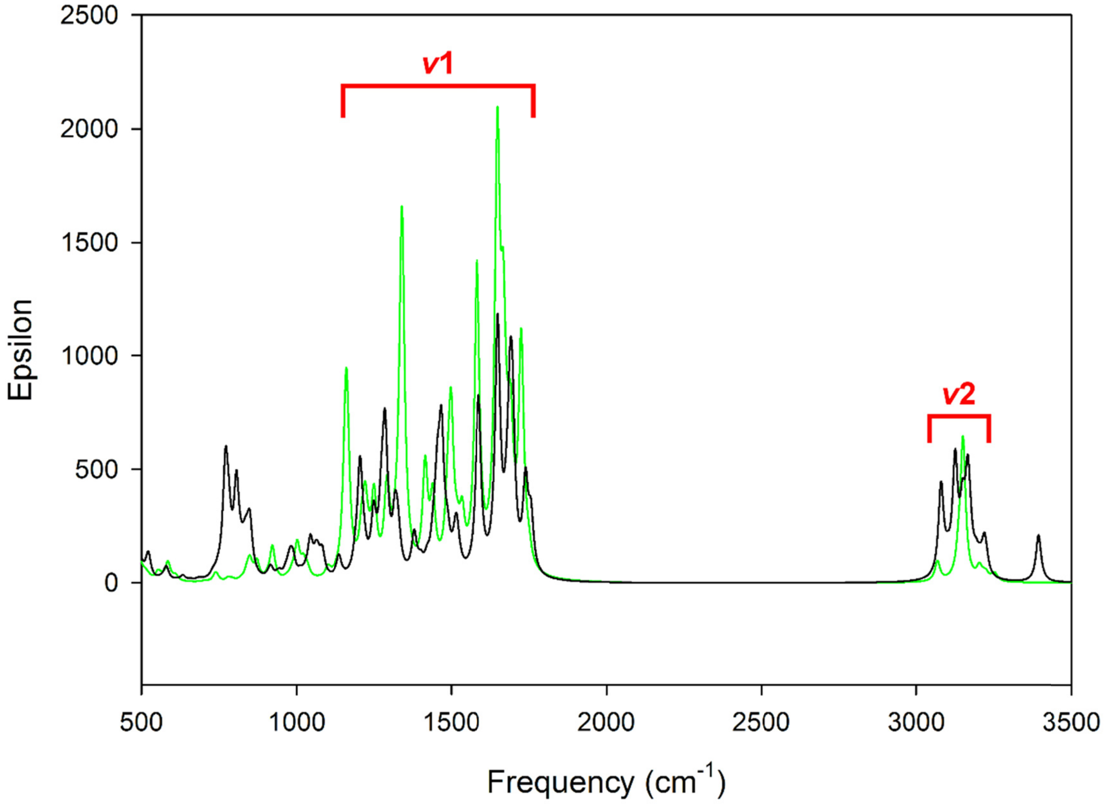

Figure S6. The FTIR spectra of (A) curcumin and (B) curcumin with selenite.

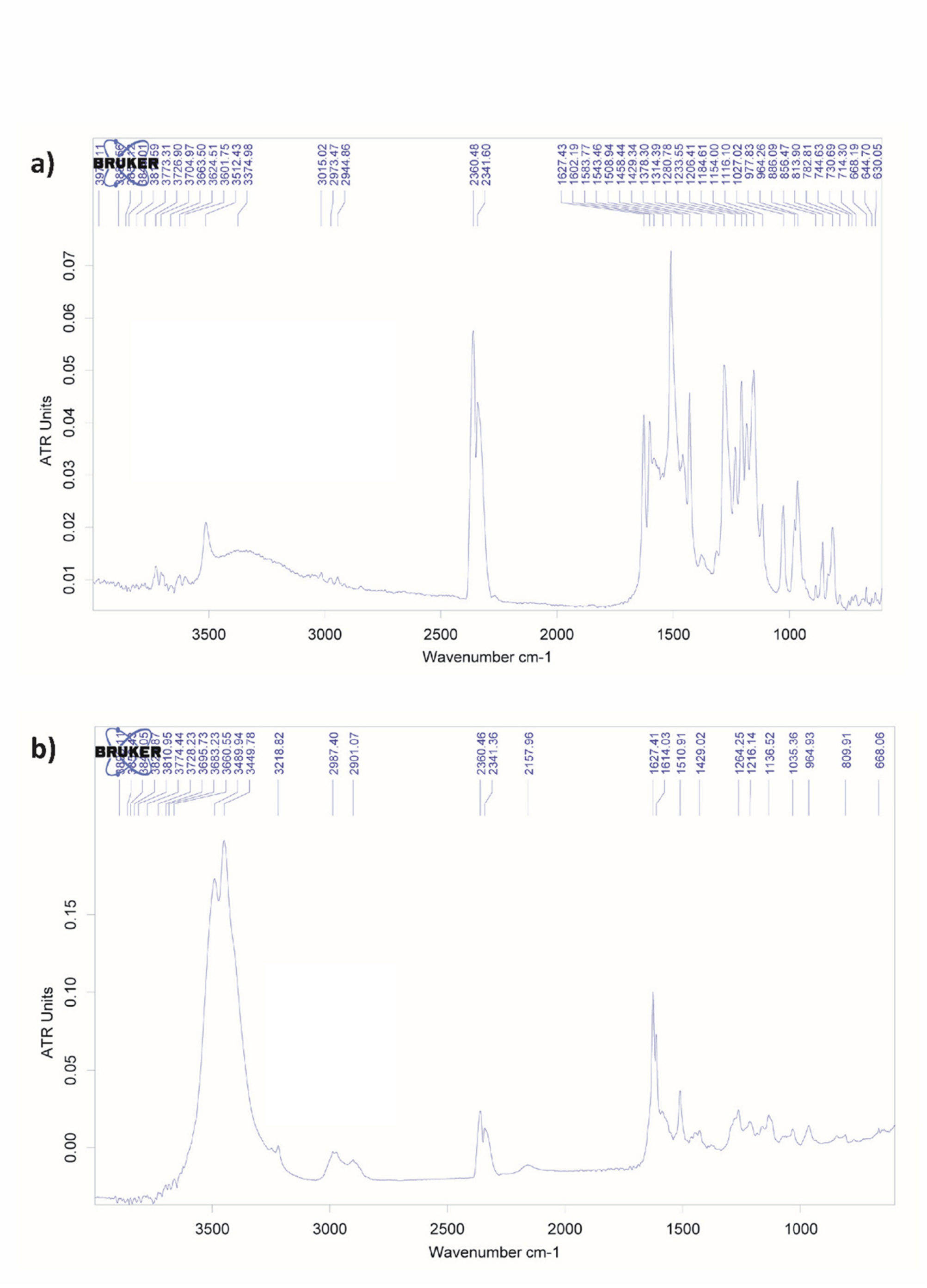

Figure S7. The FTIR spectra of (A) didemethylated curcumin and (B) didemethylated curcumin with selenite.

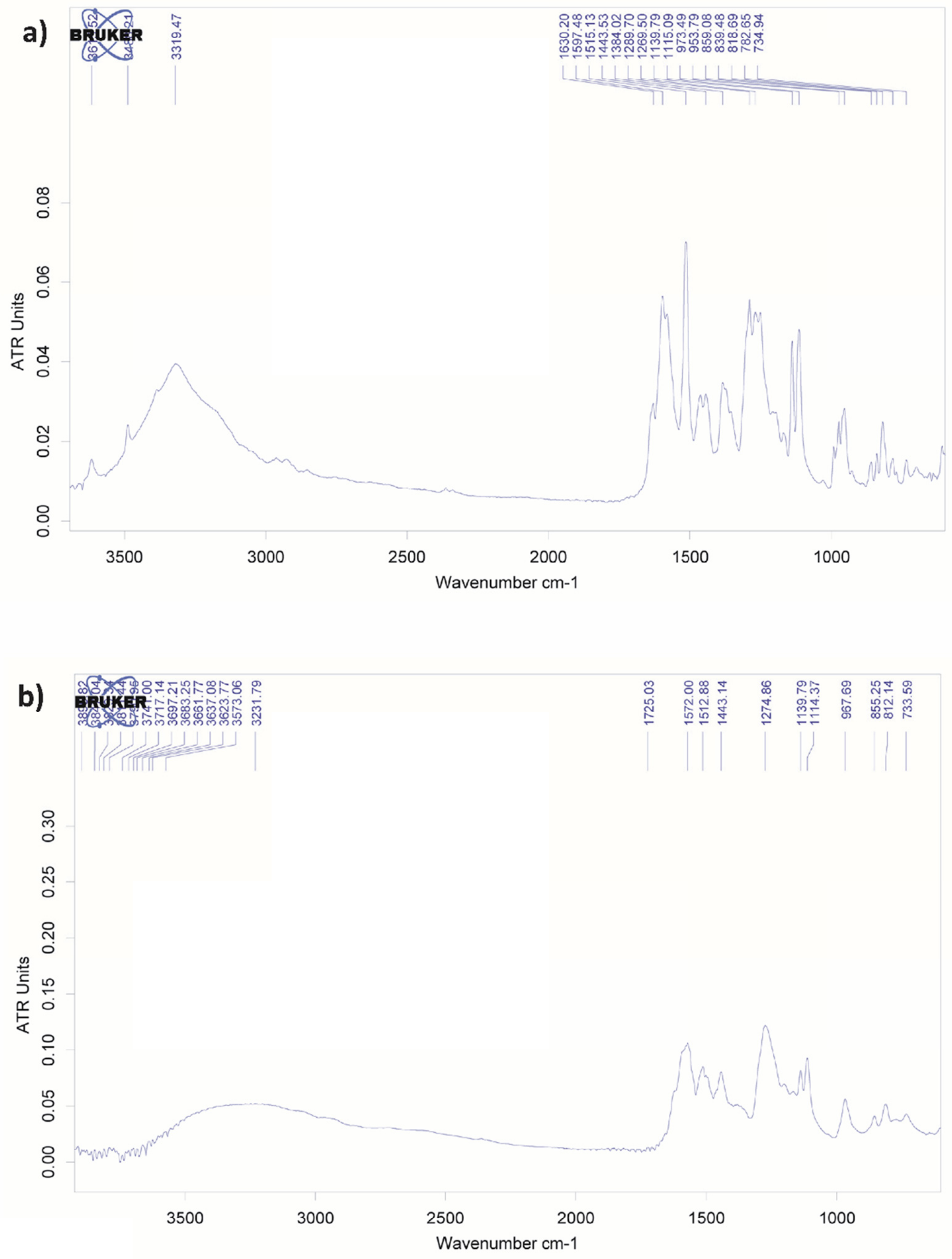

Figure S8. H^1-NMR spectra for curcumin (6 mM) with various ratios of curcumin/selenite in ACN-d_3/D_2O(3/7). Curcumin/selenite : (a) 1/32, (b) 1/16, (c) 1/8, (d) 1/4, (e) 1/2, (f) 1/1, and (g) 1/0.

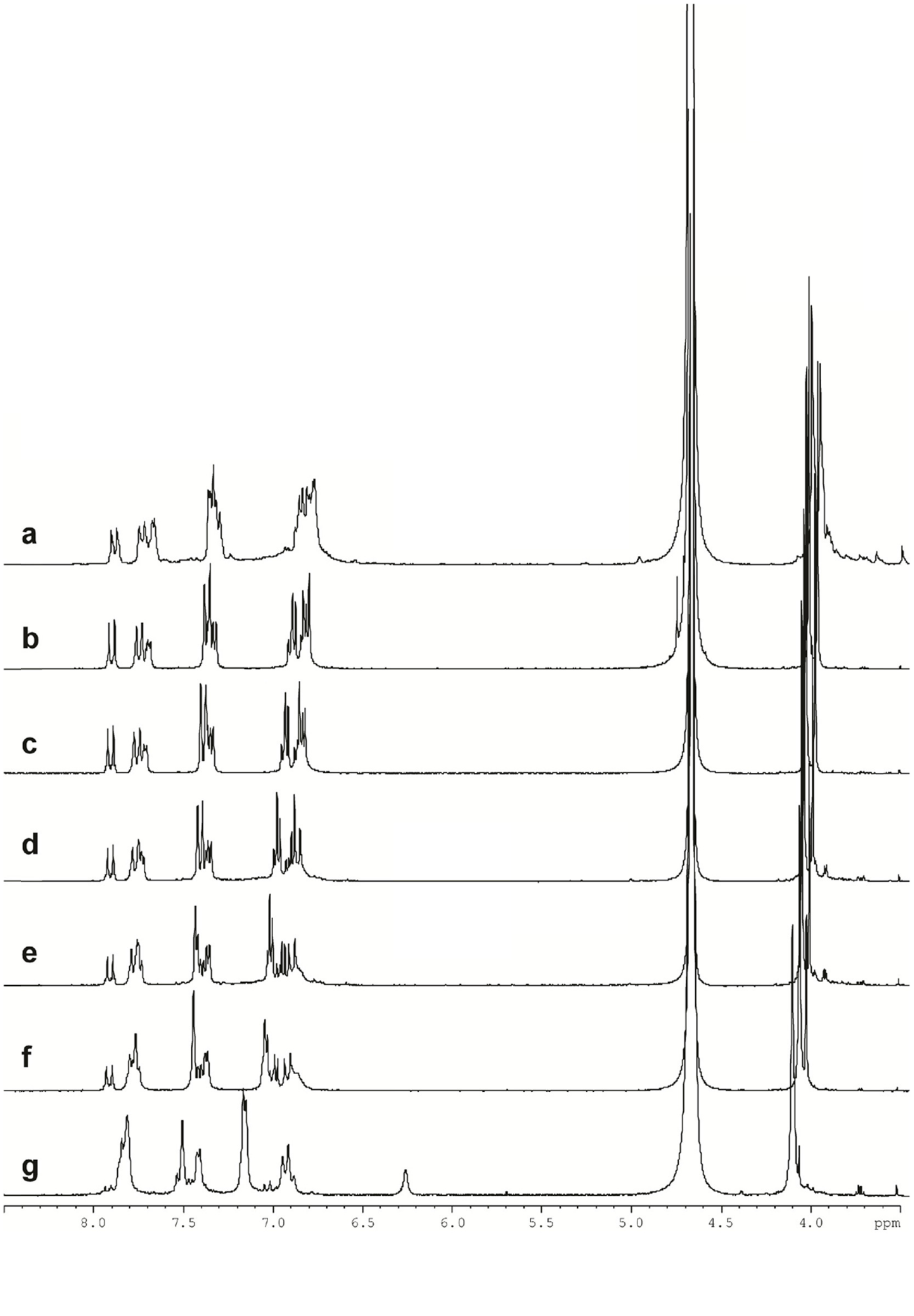

Figure S9. H^1-NMR spectra for didemethylated curcumin (6 mM) with various ratios of DCCM/selenite in methanol/ACN-d_3/D_2O(0.5:3:6.5). DCCM/selenite : (a) 1/32, (b) 1/16, (c) 1/8, (d) 1/4, (e) 1/2, (f) 1/1, and (g) 1/0.

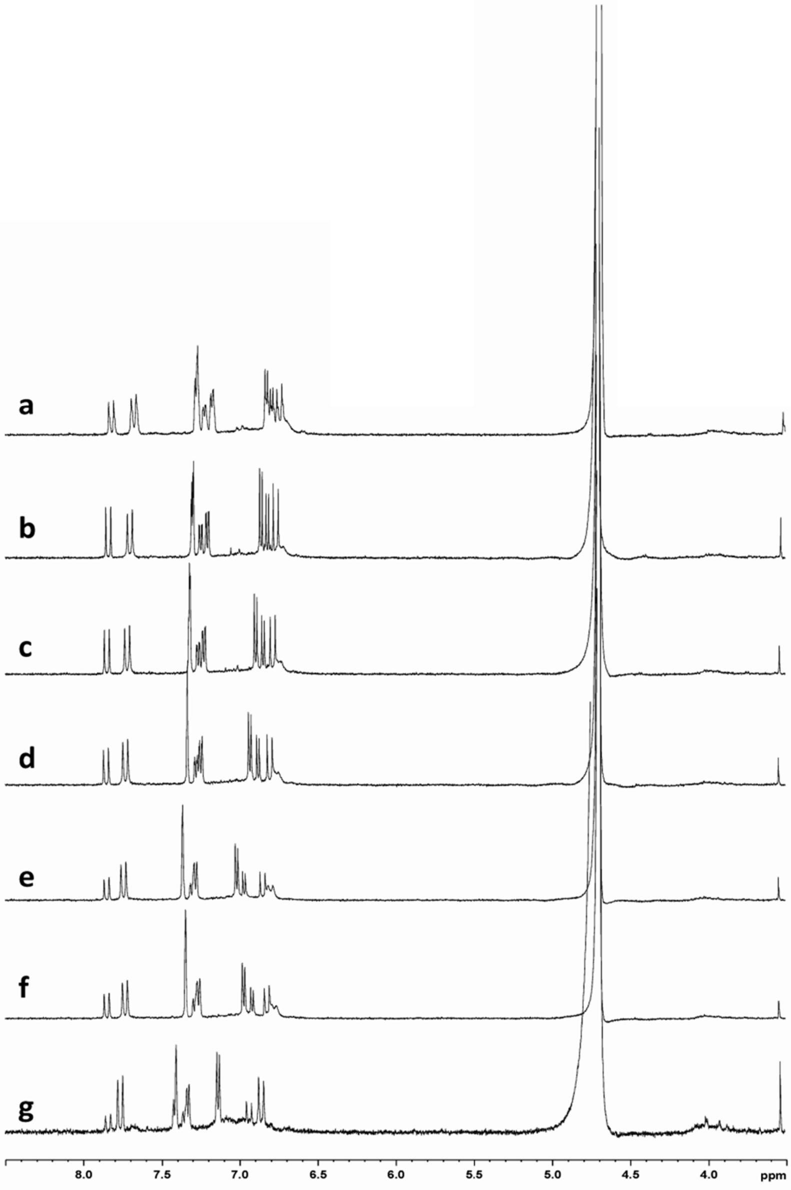

Basis Set Superposition Error (BSSE) and the Selection of Basis Set

In binding energy ($E_{binding}$) study of CCM/SeO_3^{2-} adduct (anion-neutral system), the unexpected result that $E_{binding}$ of 6-31G(d) are larger ~20 kcal/mol than 6-31+G(d). Because the reducing extant of BSSE accompanies with basis set extension, especially the adding diffuse functions is definitely beneficial effect in anion-neutral system.[1,2] On the other hand, BSSE and solvent effect are independent procedure, and they aren't executed at the same time. Therefore CS-1, CS-4, CS-7 and CS-10, which own largest $E_{binding}$ for A ~ C conformers, were used to to minimize BSSE with changing basis sets.

In table S2, the BSSE of 6-31G and 6-31+G(d) are ~30 and ~10 kcal/mol individually, and the corrected $E_{binding}$ are very close. Unfortunately, we didn't obtain BSSE of 6-311+G(2df,2p) (the geometries optimized by 6-31+G(d) were used), because SCF convergence is difficult and execution cost of quadratically convergent SCF procedure (keyword is SCF=QC) is too expensive. But the uncorrected $E_{binding}$ obtained by 6-311+G(2df,2p) are close to corrected $E_{binding}$ (only upper ~4 kcal/mol). Although slight effect appear by using these basis set for CCM monomer (see table S1), the interaction between CCM (or DCCM) and SeO_3^{2-} is key point in this research. Thus M06-2X/6-311+G(2df,2p)//M06-2X/6-31+G(d) was used to carries out energy parameters with or without solvent effect, and this method also for electronic potential plots and NBO analysis.

Reference

1 Alagona, G., Ghio, C. & Tomasi, J. Effect of counterpoise corrections on the components of the interaction energy in the formate-, acetate-, and phosphate-water dimers: a study of basis set effects. *J. Phys. Chem.* **93**, 5401-5410 (1989).

2 Alagona, G. & Ghio, C. The effect of diffuse functions on minimal basis set superposition errors for H-bonded dimers. *J. Comput. Chem.* **11**, 930-942 (1990).

3 Alagona, G., Ghio, C., Latajka, Z. & Tomasi, J. Basis set superposition errors and counterpoise corrections for some basis sets evaluated for a few $X^{-}\cdots$ M dimers. *J. Phys. Chem.* **94**, 2267-2273 (1990).
